# Endocranial volume is heritable and is associated with longevity and fitness in a wild mammal

**DOI:** 10.1098/rsos.160622

**Published:** 2016-12-14

**Authors:** C. J. Logan, L. E. B. Kruuk, R. Stanley, A. M. Thompson, T. H. Clutton-Brock

**Affiliations:** 1 Department of Zoology, University of Cambridge, Cambridge, UK; 2 Institute of Evolutionary Biology, University of Edinburgh, Edinburgh, UK; 3 Division of Evolution, Ecology and Genetics, Research School of Biology, The Australian National University, Canberra, Australia

**Keywords:** quantitative genetics, endocranial volume, life history, longevity, fitness, fecundity

## Abstract

Research on relative brain size in mammals suggests that increases in brain size may generate benefits to survival and costs to fecundity: comparative studies of mammals have shown that interspecific differences in relative brain size are positively correlated with longevity and negatively with fecundity. However, as yet, no studies of mammals have investigated whether similar relationships exist within species, nor whether individual differences in brain size within a wild population are heritable. Here we show that, in a wild population of red deer (*Cervus elaphus*), relative endocranial volume was heritable (*h*^2^ = 63%; 95% credible intervals (CI) = 50–76%). In females, it was positively correlated with longevity and lifetime reproductive success, though there was no evidence that it was associated with fecundity. In males, endocranial volume was not related to longevity, lifetime breeding success or fecundity.

## 1. Background

Across mammalian species, brain size varies widely, both in absolute terms (e.g. in weight or volume) and in relative terms (brain size accounting for overall body size, hereafter referred to as ‘relative brain size’; see [1] for a review). This variation is thought to be a consequence of interspecific differences in the relative benefits and costs of a larger brain size. Species with larger relative brain sizes may have increased cognitive abilities, enabling them to adapt to environmental changes more effectively than species with smaller brains [2,3], and may also have longer lifespans (e.g. [2,4]). However, larger brain sizes may also have disadvantages that constrain the evolution of brain size: for example, the energetic costs of brain maintenance are likely to increase with brain size (e.g. [5–9]), developmental periods may be longer and fecundity may be reduced (e.g. [9–11]). Across taxonomically diverse samples of mammalian species, relative brain size is generally positively correlated with longevity and negatively with fecundity (measured as annual reproduction or reproductive rate [9,11]), supporting the suggestion that there are both costs and benefits associated with increases in relative brain size [2–4,9–11]. However, relationships between brain size and life-history parameters vary between taxonomic groups: for example, there is no relationship between relative brain size and longevity in strepsirhine primates [2].

To date, we know little about the genetic and non-genetic determinants of variation in relative brain size in wild populations experiencing natural conditions, or whether relative brain size is associated with variation in life-history traits. While interspecific relationships between relative brain size and life-history parameters have been extensively investigated, there have been few attempts to measure intraspecific differences in brain size within populations; to determine whether these differences are related to individual differences in life-history parameters; or to relate such differences to measures of fitness. To date, empirical estimates of variation in brain size and its heritability in non-human mammals have been limited to laboratory and other non-natural conditions and include laboratory mice [12,13], laboratory rats [13], rhesus macaques (*Macaca mulatta* [14]), baboons (*Papio hamadryas* [15]), vervet monkeys (*Chlorocebus aethiops sabaeus* [16]) and chimpanzees (*Pan troglodytes* [17]). It is not yet known whether intraspecific differences in relative brain size in wild mammals are heritable [18], though a recent study on three-spined sticklebacks (*Gasterosteus aculeatus*) confirmed that individual differences in brain size have a heritable basis [19]. Furthermore, a selection experiment on relative brain mass in guppies (*Poecilia reticulata*) showed a significant realized heritability for relative brain mass, and also found a negative relationship between relative brain mass and the number of offspring produced [20].

In this study, we investigate whether individual differences in brain size in a naturally regulated population of wild red deer (*Cervus elaphus*) that has been studied for over 40 years are heritable and whether there are consistent relationships between relative brain size, longevity, fecundity and fitness in the same population. Our total sample included more than 1300 individuals, including both sexes, which have been genotyped to generate a pedigree spanning seven generations [21–23]. We measured the endocranial volumes (a proxy for brain size [24,25]) of skulls to: (i) estimate the heritability of individual differences in endocranial volume using pedigree information to run a quantitative genetic animal model analysis [26] for all-ages and adults-only, and (ii) assess selection pressures by investigating whether variation among individuals was consistently related to differences in their longevity, fecundity and lifetime breeding success (LBS).

## 2. Material and methods

### 2.1. The dataset

We used the long-term dataset from research on a study population of red deer in the North Block of the Isle of Rum, Scotland, which includes data on pedigree, social dominance, habitat use and lifetime breeding records for 4159 individuals since 1972 [21,22]. We used endocranial volume, which is an established proxy for actual brain size within bird species [24] and across mammal species [25]. Moreover, much of the interspecies literature is based on endocranial volumes (e.g. [1,9,25]), and our use of this measure provides a valid comparison with other studies of relative brain size.

### 2.2. Endocranial volume measurement

Our data are derived from the cleaned skulls of 1314 recognizable individuals whose entire life histories had been monitored in the course of a long-term study of wild red deer on the Isle of Rum [21]. We measured endocranial volume in April and May 2014 by pouring 2 mm diameter glass beads through the foramen magnum into the cranium until full, and then pouring the beads out into a graduated cylinder where the volume was recorded in millilitres. This method was previously validated as an accurate measure of endocranial volume in red deer by comparing the estimates it gave for 33 skulls with volumetric measures obtained from computerized tomography scans of the same skulls [27].

### 2.3. Relative endocranial volume

Endocranial volume is commonly correlated with body size (e.g. [28,29]): in our sample of animals it was significantly correlated with jaw length (see the electronic supplementary material, table S1). To generate a measure of relative brain size, and also because measures of body size have been shown to be heritable in our population (jaw length heritability *h*^2 ^= 0.52 female, *h*^2 ^= 0.60 male [30]) and to be associated with survival and reproduction [31,32], we controlled for effects of variation in body size in our analyses of the correlates of endocranial volume. Our analyses used jaw length as an indicator of overall body size as this was the morphological measure for which we had the largest sample size, and investigation showed that it was significantly correlated with skull length and hind leg length (see the electronic supplementary material, table S1), indicating that any of these three parameters could be used as estimates of size. We, therefore, accounted for body size by including jaw length as a fixed effect in quantitative genetic models in which endocranial volume was the dependent variable (see Animal model analyses; see the electronic supplementary material, table S2 for descriptions of all modelled variables). Outputs of these models consequently describe associations with relative endocranial volume. In models exploring the contribution of endocranial volume to variation in longevity, fecundity and fitness (see Selection analyses), we used endocranial volume and jaw length as fixed effects to determine whether endocranial volume had significant effects in addition to the effects owing to body size. As there are multiple ways to model contributions of body size and endocranial volume, we also ran two alternate variations of the selection models where fixed effects included: (i) only *relative* endocranial volume, defined as the residuals of a linear model of endocranial volume against jaw length, and (ii) both relative endocranial volume and jaw length to model the general effect of body size. The results from these models are shown in the electronic supplementary material, table S3 and are qualitatively similar to the main models.

### 2.4. Age-related variation in endocranial volume

We classified individuals as juveniles or adults based on the age at which absolute endocranial volume stopped increasing. Although absolute endocranial volume reached an asymptote (i.e. the slope was not significantly different from zero) at 2 years of age for females and at 3 years of age for males, to have a common categorization for both sexes, we defined ages 3+ as adults for both sexes; 3 years is also the youngest age at which any female had offspring in our dataset (see further details in the electronic supplementary material, Further analyses of age-related variation in endocranial volume section).

### 2.5. The pedigree

The pedigree was derived from field observations of maternal identity and from microsatellites and single nucleotide polymorphism genotyping for maternities and paternities (see [23] for full details). The pedigree was pruned to those individuals for which we had endocranial volume data and their relevant relatives and included 1715 individuals (1241 maternities and 1088 paternities), spanning seven generations (summary statistics are given in the electronic supplementary material, table S4 and figure S1; R package: pedantics, functions: drawPedigree and pedStatSummary(pedigreeStats) [33]).

### 2.6. Mixed-model analyses: determining reduced models

We modelled endocranial volume using mixed models. Absolute endocranial volume was normally distributed, therefore, models were fitted with a Gaussian error structure and identity link. We first ran full models with all variables of interest using general linear mixed models (GLMMs; R v. 3.2.1, package: lmerTest, function: lmer [34]) to determine which fixed-effect variables to include in the quantitative genetic animal models. Full models were composed of absolute endocranial volume as the response variable, with sex, age, birth weight, birth date, jaw length (therefore, outputs referred to relative endocranial volume), mother's age at parturition, mother's jaw length, mother's reproductive status and mother's location during pregnancy as fixed effects, and birth year and mother's ID as random effects (see the electronic supplementary material, table S2 for a complete list of variables, electronic supplementary material, table S5 for full model outputs and electronic supplementary material, table S1 for correlations between selected explanatory variables). Fixed effects that were significant (*p* < 0.05, using maximum likelihood, ML) were retained in the model. The Akaike information criterion (AIC) at convergence was examined when adding and removing random effects (using restricted ML). If removing a random effect decreased the AIC value, that variable was dropped from the model. Sample sizes varied between models because of missing values for explanatory variables. We ran two sets of models: all-ages (*n* = 873) to investigate the effects on endocranial volume throughout the lifespan and adult-only (*n* = 561) to investigate effects relevant only to the adults. In the all-ages model, we grouped adults as one factor level (ages 3+), while the adult-only model had age as a continuous variable (range: 3–20 years of age). We conducted a separate analysis to determine whether dominance rank should be included in the reduced models and found that it was not a significant variable in the full model, therefore, it was not used in the animal models (see the electronic supplementary material, Dominance rank section for details).

### 2.7. Animal model analyses

We used an ‘animal model’ [35] to further partition variance in endocranial volume into heritable and non-heritable components. We estimated additive genetic variance (*V*_a_) and the amount of variance explained by maternal effects (*V*_m_, mother's ID), birth year effects (*V*_b_, birth year) and the residual variance (*V*_r_) by fitting an animal model using MCMCglmm in R [36]. The animal model included fixed effects as determined by the model reduction process using lmerTest (see Mixed-model analyses: determining reduced models). To ensure model convergence, the number of iterations and/or the length of the thinning intervals were increased until autocorrelations between samples were less than 0.10 [37]; this required the following model settings: number of iterations = 3 million, burn-in = 1.2 million, thinning interval = 2000 (all-ages model), and number of iterations = 5 million, burn-in = 3 million, thinning interval = 3000 (adult-only model). The priors for each variable in both models were: *V* = 1, *n* = 0.002. Autocorrelations for the random effects in the adult-only model had seven values between 0.10 and 0.15, indicating that it converged to a large degree. We report the posterior mode of the Markov Chain Monte Carlo (MCMC) distribution of estimates of each variance component, and of the respective proportions of total variance represented by each component. Note that these modes of proportions may not sum to exactly 100%. Owing to sampling covariances, the mode of the posterior distribution of a function of parameters is not necessarily equal to the same function applied to the posterior modes of those parameters. For example, the mode of the posterior distribution of the heritability of a trait is not exactly the ratio of the posterior modes of the additive and phenotypic variance components.

### 2.8. Selection analyses: models of fitness, longevity and fecundity

We investigated selection on endocranial volume by models of longevity, fecundity and lifetime fitness, where fitness was estimated as LBS (the total number of offspring produced) and or lifetime reproductive success (LRS; the total number of offspring surviving past 1 year of age). Analyses of LRS were restricted to females because males are not involved in offspring care. Deer that died because they were shot were excluded from the fitness and longevity analyses, but not from fecundity analyses, which were based on annual rather than lifetime reproductive measures.

To determine whether LBS or LRS were associated with absolute endocranial volume, generalized linear models (GLMs) were carried out on non-shot adult (3+ years of age) males and females separately in R (females: package: stats, function: glm [38]; males: package: MASS, function: glm.nb [39,40]). Models consisted of LBS or LRS as the response variable and endocranial volume and jaw length as fixed effects (females: the LBS model was overdispersed, therefore, a quasi-Poisson family was used with a log link; the LRS model had a Poisson family with a log link; males: negative binomial error structure with a log link).

GLMs were used to investigate the relationship between absolute endocranial volume and longevity (response variable: age at death), and absolute endocranial volume and fecundity (as the response variable) in adult females and males. Longevity models had absolute endocranial volume and jaw length as explanatory variables for non-shot deer. The male GLM had a Poisson family with a log link, whereas the female GLM used a quasi-Poisson family with a log link because it was overdispersed. Fecundity models had absolute endocranial volume and jaw length as explanatory variables and included deer that died because they were shot. For the female GLM, fecundity was the proportion of years she gave birth from her first reproduction to death because females have offspring relatively consistently across their lifespan, and the model had a binomial distribution with a logit link. For the males, fecundity was modelled using a GLMM of the repeated measures of annual breeding success (total number of offspring sired each year) and was restricted to data from males at the peak of their annual breeding success (ages 8–12 [22] GLMM with ID as random effect; negative binomial distribution with a log link, package: glmmADMB, function: glmmadmb [41]).

We also analysed two additional female life-history traits: firstly an additional measure of fecundity, the age at first reproduction, and secondly an additional measure of longevity, the number of years from first reproduction to death (effectively, the length of the breeding lifespan). Absolute endocranial volume and jaw length were fitted as fixed effects in these models, and both used Poisson distributions with a log link. These additional analyses were not carried out on males because their breeding success primarily occurs during ages 8–12 [22].

## 3. Results

### 3.1. Age and sex differences in endocranial volume

**Figure 1.**
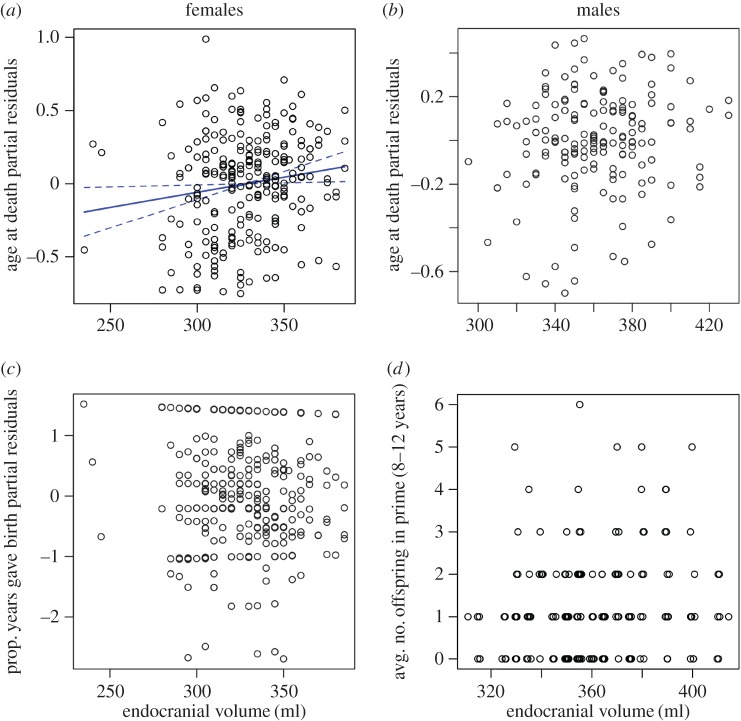
Adult (3+ years) longevity (*a*,*b*) and fecundity (*c*,*d*) plotted against absolute endocranial volume (solid line = line of best fit, dashed lines = standard errors; see table 3 for model outputs). Panels (*a*–*c*) are partial residual plots where the *y*-axis consists of the residuals (i.e. age ∼ endocranial volume) plus the predictor term (endocranial volume) to show the part of the response variable that is not explained by other explanatory variables (i.e. jaw length). (*d*) Only the relationship between the response and explanatory variable is shown because random effects impede the ability to construct partial residual plots. Females with larger endocranial volumes lived longer (*a*; excluding deer that died because they were shot), but did not reproduce at lower rates from the time they first reproduced until they died (*c*; including deer that died of any cause), whereas there was no correlation between endocranial volume and longevity (*b*) or fecundity (*d*) for males.

There were substantial differences within and between age and sex classes in absolute endocranial volume (coefficients of variance = 7.36–12.12; electronic supplementary material, figure S2 and table S6). There were also differences among adults within and between sex classes in relative endocranial volume (figure 1*a*,*b*). When analysing the full dataset of all ages together, we found that relative endocranial volume was larger in males, increased with age and was positively correlated with birth weight (table 1).

**Table 1.** Results of the animal model analyses (which include only those variables identified by the reduced models) of absolute endocranial volume (millilitres). (Model 1: all-ages, model 2: adult-only (ages 3+). We fitted Bayesian MCMC animal models using the software MCMCglmm (see ‘Animal model analyses' section in Material and methods). CI, 95% Bayesian credible intervals; n.a., not applicable. Model 1 intercept gives the value for females at age 0 with mother's location at parturition being intermediate; model 2 intercept gives the value for females with mother's reproductive status as milk/winter.)

variable	effect	95% CI	*p*-value
model 1: all-ages (*n* = 873)			
intercept	86.89	63.80–109.92	<**0**.**001**
males	14.39	11.43–17.70	<**0**.**001**
age 1	5.60	−0.59 to 11.69	0.09
age 2	16.17	7.49–26.88	**0**.**004**
ages 3+	17.16	6.21–27.86	<**0**.**001**
jaw length	0.79	0.67–0.92	<**0**.**001**
birth weight	2.71	1.51–4.00	<**0**.**001**
mother's location at parturition			
Laundry greens	−1.38	−9.23 to 5.91	0.72
Mid glen	−2.79	−9.42 to 4.12	0.43
North glen	−1.72	−7.54 to 3.65	0.54
South glen	−4.45	−12.53 to 3.28	0.30
Shamnan Insir	−3.79	−10.91 to 2.18	0.27
random effects: posterior mean			
birth year	19.31	0.0008–39.43	n.a.
mother's ID	1.93	0.0005–10.11	n.a.
animal	346.10	254.70–445.40	n.a.
residual	172.80	114.30–231.70	n.a.
model 2: adults-only (*n* = 561)			
intercept	139.93	94.50–183.38	<**0**.**002**
males	19.19	14.57–23.92	<**0**.**002**
jaw length	0.70	0.53–0.88	<**0**.**002**
mother's reproductive status:			
naive	3.21	−1.51–8.03	0.23
summer/true yelds	7.75	3.67–11.74	<**0**.**002**
random effects: posterior mean			
mother's ID	12.97	0.0003–59.27	n.a.
animal	463.30	337.2–598.20	n.a.
residual	132.80	51.38–490.70	n.a.

### 3.2. Heritability of relative endocranial volume

Relative endocranial volume was highly heritable (63%; 95% Bayesian credible intervals (CI) = 50–76%), with most of the remainder of the variance accounted for by residual variance (29%, CI = 19–44%) and almost no variance in maternal (0.003%, CI = 0.00008–2%) or birth year (3%, CI = 0.0001–7%) effects (table 2). When the analysis was restricted to adults (ages 3+), the heritability of relative endocranial volume was even higher (79%, CI = 61–90%), with most of the rest of the variance being residual (21%, CI = 7–37%) and almost no maternal effects (0.02%, CI = 0.00005–10%; table 2; birth year effects were not fitted because this was not a factor in the reduced model). Endocranial volume varied with mother's reproductive status, which could indicate her maternal investment for the current offspring (table 1).

**Table 2.** Posterior modes and 95% credible intervals (CI) for each variance component for endocranial volume (millilitres) from the animal model analyses. (*V*_a_, additive genetic variance; *V*_b_, birth year effect variance; *V*_m_, maternal variance; *V*_r_, residual variance. Variance components are followed by their 95% CI.)

source	variance components (95% CI)	per cent of total (95% CI)
model 1: all-ages		
*V*_a_	364.50 (254.68–445.40)	63 (50–76)
*V*_b_	21.51 (0.0008–39.43)	3 (0.0001–7)
*V*_m_	0.02 (0.0005–10.11)	0.003 (0.00008–2)
*V*_r_	164.03 (114.26–231.73)	29 (19–44)
model 2: adult-only		
*V*_a_	443.36 (337.19–598.20)	79 (61–90)
*V*_m_	0.14 (0.0003–59.27)	0.02 (0.00005–10)
*V*_r_	116.79 (51.38–218.99)	21 (7–37)

### 3.3. Endocranial volume and longevity

Adult females with larger endocranial volumes had significantly longer total lifespans (figure 1*a* and table 3) and longer lifespans from age at first reproduction until death, after accounting for body size effects (electronic supplementary material, figure S3; table 3). By contrast, there was no significant correlation between endocranial volume and longevity in males (table 3).

**Table 3.** Longevity and fecundity associations with absolute endocranial volume: results from models for adults (3+ years of age). (Longevity models exclude shot deer (*n* = 241 females, *n* = 167 males), whereas fecundity models include shot deer (*n* = 316 females, *n* = 64 males). As with the main female fecundity measure in the text (proportion of years gave birth), the additional measure of fecundity (described in Selection analysis; models of fitness, fecundity and longevity section) of age at first reproduction did not vary with absolute endocranial volume.)

variable	estimate	s.e.	*z*	*p*-value
females				
longevity: age at death				
* *intercept	−1.22	0.54	−2.25	**0**.**03**
* *endocranial volume	0.002	0.0009	2.30	**0**.**02**
* *jaw length	0.01	0.002	5.47	<**0**.**001**
longevity: number of years from first reproduction to death				
* *intercept	−3.57	0.53	−6.76	<**0**.**001**
* *endocranial volume	0.003	0.0009	3.60	<**0**.**001**
* *jaw length	0.02	0.002	8.63	<**0**.**001**
fecundity: proportion of years gave birth (first reproduction to death)				
* *intercept	1.31	1.25	1.05	0.30
* *endocranial volume	−0.002	0.002	−1.00	0.32
* *jaw length	0.0007	0.005	0.15	0.88
fecundity: age at first reproduction				
intercept	1.009	0.71	1.42	0.16
endocranial volume	0.0001	0.001	0.11	0.91
jaw length	0.001	0.003	0.51	0.61
males				
longevity: age at death				
intercept	0.40	0.68	0.59	0.55
endocranial volume	0.001	0.001	1.13	0.26
jaw length	0.008	0.002	3.61	<**0**.**001**
fecundity: number of offspring per year in prime (8–12 years)				
intercept	2.12	3.04	−0.79	0.43
endocranial volume	0.006	0.004	1.42	0.16
jaw length	0.0008	0.009	0.08	0.93
* *age 9	0.17	0.19	0.90	0.37
* *age 10	0.21	0.21	1.02	0.31
* *age 11	0.51	0.21	2.42	0.02
* *age 12	0.59	0.29	2.04	0.04
* *random effect: ID	variance: 0.20	s.d.: 0.45		

### 3.4. Endocranial volume and fecundity

There was no indication that large endocranial volumes were negatively related to fecundity or breeding success in either sex or to female age at first reproduction (figure 1 and table 3; electronic supplementary material, figure S3). After body size effects were accounted for, females with larger endocranial volumes were as likely to give birth in any year of their lifespan as females with smaller endocranial volumes (table 3), and the proportion of years from age at first reproduction until death in which females produced offspring was not correlated with endocranial volume (table 3 and figure 1). After body size effects were accounted for, endocranial volume in males did not correlate with annual breeding success in their prime reproductive years (8–12 years of age; table 3).

### 3.5. Endocranial volume and fitness

**Figure 2.**
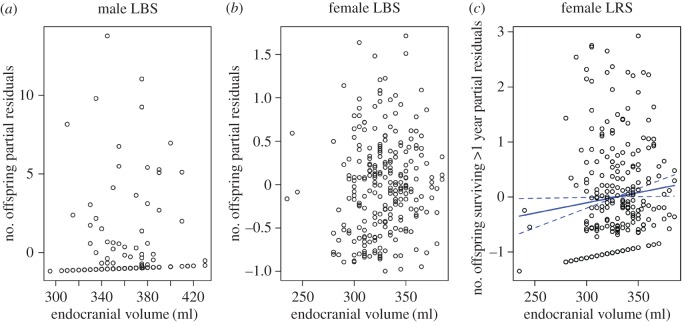
Partial residual plots: lifetime breeding (*a*,*b*) and reproductive success (*c*) and absolute endocranial volume for adults (ages 3+, solid line = line of best fit, dashed lines = standard errors). The relationship in (*c*) was statistically significant (table 4). The *y*-axis consists of the residuals (i.e. fitness∼endocranial volume) plus the predictor term (endocranial volume) to show the part of the response variable that is not explained by other explanatory variables (i.e. jaw length).

After body size effects were accounted for, there was no association between endocranial volume and LBS in either sex (table 4 and figure 2) but endocranial volume was positively associated with female LRS (table 4 and figure 2; see the electronic supplementary material, figure S4 for absolute endocranial volume without accounting for jaw length). This effect was probably owing to the significant positive relationship between female endocranial volume and longevity (see above) as there was no significant association between juvenile survival rate and endocranial volume (electronic supplementary material, table S7; see the GLM of binomial proportions with LRS as a proportion of LBS as the response variable and endocranial volume and jaw length as explanatory variables, using a binomial distribution with a logit link).

**Table 4.** Fitness associations with absolute endocranial volume: results from the lifetime breeding success (males and females) and lifetime reproductive success (females) models for adults (3+ years of age, EV = endocranial volume, means in millilitres for EV and millimetres for jaw length, a *t*-statistic for female LBS and a *z* statistic for other models were automatically determined by R and depended on whether the standard errors were known, bold and italic = significant). (GLMs consisted of LBS or LRS as the response variable and endocranial volume and jaw length as fixed effects. The male LBS model had a negative binomial distribution and a log link; the female LBS model had a quasi-Poisson distribution with a log link because it was overdispersed; and the female LRS model had a Poisson distribution with a log link.)

		absolute endocranial volume				jaw length			
model	*N*	estimate	s.e.	*t/z*	*p*-value	estimate	s.e.	*t/z*	*p*-value
LBS male	166	0.003	0.009	0.31	0.76	0.09	0.02	4.10	<***0***.***001***
LBS female	244	0.001	0.001	1.02	0.31	0.02	0.003	4.96	<***0***.***001***
LRS female	244	0.004	0.002	2.17	***0***.***03***	0.02	0.004	5.61	<***0***.***001***

## 4. Discussion

We found that individual differences in endocranial volume in red deer were substantial, with coefficients of variation in the range of 7–12 (electronic supplementary material, table S6). Individual differences in relative endocranial volume were highly heritable, generating the highest value of heritability (63%) for any morphometric trait yet investigated in this population [22,30]. In females, endocranial volume was positively associated with longevity and fitness, but not with fecundity, after accounting for body size. However, the sizes of these effects were relatively small (see figures 1 and 2 and estimates in tables 3 and 4). In males, endocranial volume was not significantly related to longevity, fecundity or fitness after accounting for body size.

Comparative analyses of species differences in relative brain size have suggested that increases in relative brain size may generate benefits to survival and longevity (e.g. [2–4]) but may reduce fecundity (e.g. [9,11]). The positive correlation between endocranial volume and longevity in females is consistent with this suggestion, though we cannot tell whether there is any causal link between the parameters. By contrast, we did not find any evidence that endocranial volume negatively correlated with fecundity, whereas it does in interspecies comparisons [9,11]. If large brains are costly, perhaps these costs are absorbed by other traits such as having a shorter gut length or reduced immunity, as in guppies [20,42] and across wild cichlid species [43], but see Walsh *et al.* [44] for no relationship with gut length in killifish (*Rivulus hartii*).

In conjunction, evidence that relative brain size in females is significantly correlated with LRS indicates that it is under selection. As endocranial volume was not significantly correlated with indices of female fecundity after accounting for body size, the association between endocranial volume and LRS was probably owing to the positive correlation between female endocranial volume and longevity. Several possible explanations for positive correlations between relative brain size and longevity have been suggested, including that larger-brained individuals (i) are better at surviving [3], (ii) have longer overall life cycles and thus more time for brain growth, or (iii) can more flexibly adjust their metabolism during times of environmental stress (e.g. by downregulating their metabolic rate or consuming alternative resources [2]). These hypotheses remain speculative owing to a lack of cognitive, brain growth and metabolic data, and our results cannot discriminate among them.

It is also unclear why there were no significant relationships between endocranial volume and longevity, fecundity or fitness in males. One possibility is that large individual differences in male expenditure on reproduction during the rut (see [21]) exert important effects on survival and longevity [45] and obscure any consistent association between brain size and longevity in males. Alternatively, perhaps male braincases are constrained by the need to grow and use antlers for combat with other males. The observation of positive selection in females but not in males might lead us to expect that females should have larger relative brain sizes than males—the opposite of the observed sexual dimorphism. We speculate that this apparent contradiction indicates the presence of unknown trade-offs or constraints shaping the evolutionary trajectories of brain size in either sex. In an analogous situation, antler size was heritable and positively associated with LBS in this population, but showed no evolutionary response over time, providing another example of the joint occurrence of heritability and selection on a trait without an evolutionary response [22].

Our results lead us to draw three main conclusions relevant to interspecies hypotheses about why brain sizes vary: (i) individuals differ in endocranial volume; (ii) relative endocranial volume is heritable; and (iii) the positive correlations between endocranial volume and longevity in females suggest that larger brain size at the individual level may confer some advantages, which translate into higher female LRS, though these benefits may be offset by costs to other components of fitness. Future research investigating the causal links between brain size and life-history traits will determine whether hypotheses generated from interspecies correlations about the costs and benefits of investing in a large brain are valid and how the relationships we found within a species are causally linked.

## Permission to carry out the fieldwork

Permission to carry out the fieldwork for this project was given by the Nature Conservancy Council and, latterly, by Scottish Natural Heritage who own the Isle of Rum, which is a National Nature Reserve.

## Supplementary Material

Additional figures, tables, and analyses Contents: -Supplementary Tables S1-S8 -Dominance ranks (Supplementary Table S8) -Supplementary Figures S1-S3 -Further analyses of age-related variation in endocranial volume (Supplementary Figure S2)

## Data accessibility

Data are available at the KNB Data Repository [46].

## References

[1] Roth G, Dicke U 2005 Evolution of the brain and intelligence. Trends Cogn. Sci. 9, 250–257. (doi:10.1016/j.tics.2005.03.005) 15866152 10.1016/j.tics.2005.03.005

[2] Allman JM, McLaughlin T, Hakeem A 1993 Brain-weight and life-span in primate species. Proc. Natl Acad. Sci. USA 90, 118–122. (doi:10.1073/pnas.90.1.118) 8419913 10.1073/pnas.90.1.118 PMC45611

[3] Sol D, Szekely T, Liker A, Lefebvre L 2007 Big-brained birds survive better in nature. Proc. R. Soc. B 274, 763–769. (doi:10.1098/rspb.2006.3765) 10.1098/rspb.2006.3765 PMC2093983 17251112

[4] González-Lagos C, Sol D, Reader SM 2010 Large-brained mammals live longer. J. Evol. Biol. 23, 1064–1074. (doi:10.1111/j.1420-9101.2010.01976.x) 20345813 10.1111/j.1420-9101.2010.01976.x

[5] Nyberg D 1971 A hypothesis concerning the larger brains of homoiotherms. Am. Nat. 105, 183–185. (doi:10.1086/282713)

[6] Harvey PH, Bennett PM 1983 Evolutionary biology: brain size, energetics, ecology and life history patterns. Nature 306, 314–315. (doi:10.1038/306314a0) 6646214 10.1038/306314a0

[7] Armstrong E 1983 Relative brain size and metabolism in mammals. Science 220, 1302–1304. (doi:10.1126/science.6407108) 6407108 10.1126/science.6407108

[8] Aiello LC, Wheeler P 1995 The expensive-tissue hypothesis: the brain and the digestive system in human and primate evolution. Curr. Anthropol. 36, 199–221. (doi:10.1086/204350)

[9] Isler K, van Schaik CP 2009 The expensive brain: a framework for explaining evolutionary changes in brain size. J. Hum. Evol. 57, 392–400. (doi:10.1016/j.jhevol.2009.04.009) 19732937 10.1016/j.jhevol.2009.04.009

[10] Deaner RO, Barton RA, van Schaik CP 2003 Primate brains and life histories: renewing the connection. In Primate life histories and socioecology (eds Kappeler PM, Pereira ME), pp. 233–265. Chicago, IL: University of Chicago Press.

[11] Isler K 2011 Energetic trade-offs between brain size and offspring production: marsupials confirm a general mammalian pattern. BioEssays 33, 173–179. (doi:10.1002/bies.201000123) 21254150 10.1002/bies.201000123

[12] Atchley WR, Riska B, Kohn LAP, Plummer AA, Rutledge JJ 1984 A quantitative genetic analysis of brain and body size associations, their origin and ontogeny: data from mice. Evolution 38, 1165–1179. (doi:10.2307/2408625) 10.1111/j.1558-5646.1984.tb05640.x 28563775

[13] Riska B, Atchley WR 1985 Genetics of growth predict patterns of brain-size evolution. Science 229, 668–671. (doi:10.1126/science.229.4714.668) 17739380 10.1126/science.229.4714.668

[14] Cheverud JM, Falk D, Vannier M, Konigsberg L, Helmkamp RC, Hildebolt C 1990 Heritability of brain size and surface features in rhesus macaques (*Macaca mulatta*). J. Heredity 81, 51–57. 10.1093/oxfordjournals.jhered.a110924 2332614

[15] Rogers J, Kochunov P, Lancaster J, Shelledy W, Glahn D, Blangero J, Fox P 2007 Heritability of brain volume, surface area and shape: an MRI study in an extended pedigree of baboons. Hum. Brain Mapp. 28, 576–583. (doi:10.1002/hbm.20407) 17437285 10.1002/hbm.20407 PMC6871350

[16] Fears SC et al. 2009 Identifying heritable brain phenotypes in an extended pedigree of vervet monkeys. J. Neurosci. 29, 2867–2875. (doi:10.1523/JNEUROSCI.5153-08.2009) 19261882 10.1523/JNEUROSCI.5153-08.2009 PMC2716293

[17] Gómez-Robles A, Hopkins WD, Schapiro SJ, Sherwood CC 2015 Relaxed genetic control of cortical organization in human brains compared with chimpanzees. Proc. Natl Acad. Sci. USA 112, 14 799–14 804. (doi:10.1073/pnas.1512646112) 26627234 10.1073/pnas.1512646112 PMC4672807

[18] Gonda A, Herczeg G, Merilä J 2013 Evolutionary ecology of intraspecific brain size variation: a review. Ecol. Evol. 3, 2751–2764. (doi:10.1002/ece3.627) 24567837 10.1002/ece3.627 PMC3930043

[19] Noreikiene K, Herczeg G, Gonda A, Balázs G, Husby A, Merilä J 2015 Quantitative genetic analysis of brain size variation in sticklebacks: support for the mosaic model of brain evolution. Proc. R. Soc. B 282, 20151008 (doi:10.1098/rspb.2015.1008) 10.1098/rspb.2015.1008 PMC4590490 26108633

[20] Kotrschal A, Rogell B, Bundsen A, Svensson B, Zajitschek S, Brännström I, Immler S, Maklakov AA, Kolm N 2013 Artificial selection on relative brain size in the guppy reveals costs and benefits of evolving a larger brain. Curr. Biol. 23, 168–171. (doi:10.1016/j.cub.2012.11.058) 23290552 10.1016/j.cub.2012.11.058 PMC3566478

[21] Clutton-Brock TH, Guinness FE, Albon SD 1982 Red deer: behavior and ecology of two sexes. Chicago, IL: University of Chicago Press.

[22] Kruuk LEB, Slate J, Pemberton JM, Brotherstone S, Guinness F, Clutton-Brock T 2002 Antler size in red deer: heritability and selection but no evolution. Evolution 56, 1683–1695. (doi:10.1111/j.0014-3820.2002.tb01480.x) 12353761 10.1111/j.0014-3820.2002.tb01480.x

[23] Huisman J, Kruuk LEB, Ellis PA, Clutton-Brock T, Pemberton JM 2016 Inbreeding depression across the lifespan in a wild mammal population. Proc. Natl Acad. Sci. USA 113, 3585–3590. (doi:10.1073/pnas.1518046113) 26979959 10.1073/pnas.1518046113 PMC4822623

[24] Iwaniuk AN, Nelson JE 2002 Can endocranial volume be used as an estimate of brain size in birds? Can. J. Zool. 80, 16–23. (doi:10.1139/z01-204)

[25] Isler K, Kirk EC, Miller JMA, Albrecht GA, Gelvin BR, Martin RD 2008 Endocranial volumes of primate species. Scaling analyses using a comprehensive and reliable dataset. J. Hum. Evol. 55, 967–978. (doi:10.1016/j.jhevol.2008.08.004) 18817943 10.1016/j.jhevol.2008.08.004

[26] Kruuk LEB 2004 Estimating genetic parameters in natural populations using the ‘animal model’. Phil. Trans. R. Soc. Lond. B 359, 873–890. (doi:10.1098/rstb.2003.1437) 15306404 10.1098/rstb.2003.1437 PMC1693385

[27] Logan CJ, Clutton-Brock TH 2013 Validating methods for measuring endocranial volume in individual red deer (*Cervus elaphus*). Behav. Process 92, 143–146. (doi:10.1016/j.beproc.2012.10.015) 10.1016/j.beproc.2012.10.015 23137587

[28] Jerison HJ 1973 Evolution of the brain and intelligence. New York, NY: Academic Press.

[29] Montgomery SH, Geisler JH, McGowen MR, Fox C, Marino L, Gatesy J 2013 The evolutionary history of cetacean brain and body size. Evolution 67, 3339–3353. (doi:10.1111/evo.12197) 24152011 10.1111/evo.12197

[30] Kruuk LEB, Clutton-Brock TH, Slate J, Pemberton JM, Brotherstone S, Guinness FE 2000 Heritability of fitness in a wild mammal population. Proc. Natl Acad. Sci. USA 97, 698–703. (doi:10.1073/pnas.97.2.698) 10639142 10.1073/pnas.97.2.698 PMC15393

[31] Albon SD, Clutton-Brock TH, Guinness FE 1987 Early development and population dynamics in red deer. II. Density-independent effects and cohort variation. J. Anim. Ecol. 56, 69–81.

[32] Kruuk LEB, Clutton-Brock TH, Albon SD, Pemberton JM, Guinness FE 1999 Population density affects sex ratio variation in red deer. Nature 399, 459–461. (doi:10.1038/20917) 10365956 10.1038/20917

[33] Morrissey MB, Wilson AJ 2010 PEDANTICS: an R package for pedigree-based genetic simulation and pedigree manipulation, characterization and viewing. Mol. Ecol. Resour. 10, 711–719. (doi:10.1111/j.1755-0998.2009.02817.x) 21565076 10.1111/j.1755-0998.2009.02817.x

[34] Kuznetsova A, Brockhoff PB, Christensen RHB 2015 lmerTest: tests in linear mixed effects models. R package. See https://cran.r-project.org/web/packages/lmerTest/index.html (accessed 30 July 2015).

[35] Wilson AJ, Réale D, Clements MN, Morrissey MM, Postma E, Walling CA, Kruuk LEB, Nussey DH 2010 An ecologist's guide to the animal model. J. Anim. Ecol. 79, 13–26. (doi:10.1111/j.1365-2656.2009.01639.x) 20409158 10.1111/j.1365-2656.2009.01639.x

[36] Hadfield JD 2010 MCMC methods for multi-response generalized linear mixed models: the MCMCglmm R package. J. Stat. Softw. 33, 1–22. (doi:10.18637/jss.v033.i02)20808728

[37] Hadfield J. 2014 MCMCglmm course notes. See http://cran.r-project.org/web/packages/MCMCglmm/vignettes/CourseNotes.pdf (accessed 30 July 2015).

[38] R Core Team. 2015 R: a language and environment for statistical computing. Vienna, Austria: R Foundation for Statistical Computing See https://www.R-project.org (accessed 1 January 2016).

[39] Ripley B, Venables B, Bates DM, Hornik K, Gebhardt A, Firth D. 2015 MASS: support functions and datasets for Venables and Ripley's MASS. R package. See https://cran.r-project.org/web/packages/MASS/index.html (accessed 1 August 2015).

[40] Venables WN, Ripley BD 2002 Modern applied statistics with S, 4th edn Oxford, UK: Springer.

[41] Skaug H, Fournier D, Nielsen A, Magnusson A, Bolker B. 2015 Generalized linear mixed models using ‘AD Model Builder’. R package. See http://glmmadmb.r-forge.r-project.org (accessed 8 September 2015).

[42] Kotrschal A, Kolm N, Penn DJ 2016 Selection for brain size impairs innate, but not adaptive immune responses. Proc. R. Soc. B 283, 20152857 (doi:10.1098/rspb.2015.2857) 10.1098/rspb.2015.2857 PMC4810857 26962144

[43] Tsuboi M, Husby A, Kotrschal A, Hayward A, Buechel SD, Zidar J, Løvlie H, Kolm N 2015 Comparative support for the expensive tissue hypothesis: big brains are correlated with smaller gut and greater parental investment in Lake Tanganyika cichlids. Evolution 69, 190–200. (doi:10.1111/evo.12556) 25346264 10.1111/evo.12556 PMC4312921

[44] Walsh MR, Broyles W, Beston SM, Munch SB 2016 Predator-driven brain size evolution in natural populations of Trinidadian killifish (*Rivulus hartii*). Proc. R. Soc. B 283, 20161075 (doi:10.1098/rspb.2016.1075) 10.1098/rspb.2016.1075 PMC4947895 27412278

[45] Lemaître J-F, Gaillard J-M, Pemberton JM, Clutton-Brock TH, Nussey DH 2014 Early life expenditure in sexual competition is associated with increased reproductive senescence in male red deer. Proc. R. Soc. B 281, 20140792 (doi:10.1098/rspb.2014.0792) 10.1098/rspb.2014.0792 PMC4150313 25122226

[46] Logan CJ, Kruuk LEB, Pemberton J, Clutton-Brock TH 2016 Endocranial volume measurements from 1078 red deer on the Isle of Rum, Scotland, 1972–2013. KNB Data Repository. (doi:10.5063/F1JW8BT9)

