# Supplementary material for: Endocranial volume is heritable and is associated with longevity and fitness in a wild mammal
Source: R Soc Open Sci. 2016 Dec 14;3(12):160622. doi: 10.1098/rsos.160622 (PMC5210687; doi:10.1098/rsos.160622)
Supplement: Additional figures, tables, and analyses Contents: -Supplementary Tables S1-S8 -Dominance ranks (Supplementary Table S8) -Supplementary Figures S1-S3 -Further analyses of age-related variation in endocranial volume (Supplementary Figure S2) [file rsos160622supp1.pdf]

SUPPLEMENTARY MATERIAL for:

Logan CJ, Kruuk L, Stanley R, Thompson A, Clutton-Brock TH. 2016. Endocranial volume is heritable and is associated with longevity and fitness in a wild mammal. *Royal Society Open Science* 3:160622. http://dx.doi.org/10.1098/rsos.160622

<u>**Contents:**</u>

Supplementary Tables S1-S8

Dominance rank (Supplementary Table S8)

Supplementary Figures S1-S4

Further analyses of age-related variation in endocranial volume (Supplementary Figure S2)

References

Supplementary Table S1. The relationships between selected variables: Pearson's product-moment correlations between endocranial volume, jaw length, skull length, back leg length, lifetime breeding success, lifetime reproductive success, and longevity (CI=confidence interval, a Bonferroni-Holm correction was applied to p-values to attempt to control for an increase in type 1 errors from conducting multiple tests on the same dataset).

**Correlation**	**Sex**	**r**	**95% CI**	**t**	**df**	**p**
Endocranial volume – jaw length	Female	0.81	0.78-0.84	32.57	547	**<0.001**
	Male	0.88	0.86-0.90	44.96	584	**<0.001**
Jaw length – skull length	Female	0.97	0.96-0.97	86.58	501	**<0.001**
	Male	0.98	0.98-0.99	112.65	460	**<0.001**
Jaw length – back leg length	Female	0.85	0.79-0.90	16.52	105	**<0.001**
	Male	0.87	0.80-0.91	15.86	82	**<0.001**
Lifetime breeding success – lifetime reproductive success	Female	0.82	0.79-0.85	33.51	546	**<0.001**
Lifetime breeding success - longevity	Female	0.94	0.93-0.95	63.20	546	**<0.001**
	Male	0.40	0.32-0.46	10.35	578	**<0.001**
Lifetime reproductive success - longevity	Female	0.78	0.74-0.81	29.02	546	**<0.001**

Supplementary Table S2. A description of all variables in the full models.

Variable	Description
Fixed effects	
Sex	Female or Male
Jaw length	An indicator of body size: Length of the jaw bone in mm from the back to the protruding piece of bone at the bottom of the front incisor
Birth weight	Weight in kg at capture was adjusted to estimate birth weight by subtracting the capture age in hours multiplied by 0.01696kg/hr [23]
Birth date	The number of days between the birth date and 1 May
Age	Age at death, estimated assuming that all individuals are born after 1 May
Maternal location during pregnancy	The study area is divided into six areas: north glen, laundry greens, south glen, mid glen, intermediate and Shamnan Insir. These serve as an indicator of habitat quality: higher quality habitat being north glen and laundry greens and the lower quality habitat being south glen, mid glen, intermediate, and Shamnan Insir [23]
Dominance rank (females)	Available only for females. Calculations from [32] (see Materials and Methods above)
Maternal dominance rank	Calculations from [32] (see Materials and Methods above)
Maternal age at parturition	The age of the mother when the calf was born calculated as above
Maternal jaw length	The jaw length (as above) of the mother as an indicator of body size
Mother's reproductive status	The mother's status was grouped into 3 categories defined by likely levels of resources available for investment based on amounts invested in offspring in a previous year (as in [21]:<br>(i) '*Low'* available investment were Milk Hind and Winter Yeld hinds because both gave birth the year before and their offspring either died in the winter (after 1 October in the previous year; winter yeld) or survived its first year (milk hind).<br>(ii) '*High'* available investment categories were Summer and True Yelds because they either gave birth the year before but the offspring did not survive its first few months (died before 1 October in the previous year; summer yeld) or had given birth at some point, but not in the previous year (true yeld).<br>(iii) High available investment but '*inexperienced'* was the Naive status where it was a female's first time giving birth.
Random effects	
Birth year	The year in which the individual was born
Mother's ID	The ID of the subject's mother
Animal ID	The ID of the subject linked to the pedigree through the "ped" function in the model

Supplementary Table S3. Fecundity and longevity models run with two variations of modeling relative endocranial volume (relEV: residuals of endocranial volume against jaw length) as fixed effects (either (a) relative endocranial volume or (b) relative endocranial volume + jaw length) show that, with two exceptions, conclusions do not differ from the models used in the text (fixed effects: endocranial volume + jaw length). The exceptions occur in the female LRS and female longevity models where, in both cases, the relative endocranial volume is not significant in the relative endocranial volume only model. This is in contrast with the other two methods of modeling relative endocranial volume, which both show this variable as significant (right side of the table below and in Tables 3 and 4). Random effects show variances and standard deviations (SD).

	(a) **Fixed effects: Relative endocranial volume (relEV)**					*(b)* **Fixed effects: Relative endocranial volume (relEV) + jaw length (JL)**				
**Model**	**Variable**	**Estimate**	**SE**	**z**	**p**	**Variable**	**Estimate**	**SE**	**z**	**p**
**Male LBS**<br>Lifetime breeding success<br>GLM negative binomial with log link	Intercept	0.79	0.25	3.17	0.002	Intercept	25.019	6.13	-4.08	<0.001
	relEV	0.008	0.009	-0.88	0.38	relEV	0.003	0.009	0.31	0.76
						JL	0.09	0.02	4.17	<0.001
**Male fecundity**<br>Number of offspring per yr in prime<br>GLMM negative binomial with log link	Intercept	-0.16	0.15	-1.04	0.30	Intercept	-2.03	2.92	-0.69	0.49
	relEV	0.005	0.004	1.26	0.21	relEV	0.006	0.004	1.42	0.16
	Age 9	0.17	0.19	0.94	0.35	JL	0.007	0.01	0.64	0.52
	Age 10	0.22	0.21	1.07	0.28	Age 9	0.17	0.19	0.91	0.36
	Age 11	0.52	0.21	2.47	0.01	Age 10	0.21	0.21	1.02	0.31
	Age 12	0.60	0.29	2.10	0.04	Age 11	0.51	0.21	2.43	0.02
	Random: ID	Variance: 0.20	SD: 0.46			Age 12	0.59	0.29	2.04	0.04
						Random: ID	0.20	0.45		
**Male longevity**<br>Age at death<br>GLM Poisson with log link	Intercept	2.33	0.03	89.96	<0.002	Intercept	-0.33	0.66	-0.50	0.62
	relEV	0.0002	0.0009	0.21	0.83	relEV	0.001	0.001	1.13	0.26
						JL	0.01	0.002	4.03	<0.001
**Female LBS**<br>GLM quasipoisson (overdispersed) with log link	Intercept	1.76	0.04	48.77	<0.001	Intercept	-2.94	0.85	-3.46	<0.001
	relEV	0.0002	0.001	0.12	0.91	relEV	0.001	0.001	1.02	0.31
						JL	0.02	0.003	5.56	<0.001
**Female LRS**<br>GLM Poisson with log link	Intercept	0.93	0.04	22.73	<0.001	Intercept	-5.80	1.00	-5.80	<0.001
	relEV	0.002	0.002	1.09	0.28	relEV	0.003	0.002	2.17	0.03
						JL	0.03	0.004	6.77	<0.001
**Female fecundity**<br>Proportion yrs gave birth (first birth to death)<br>GLM binomial with logit link	Intercept	0.88	0.05	19.02	<0.001	Intercept	1.19	1.22	0.97	0.33
	relEV	-0.002	0.002	-0.97	0.33	relEV	-0.002	0.002	-1.00	0.32
						JL	0.001	0.005	0.26	0.80
**Female longevity**<br>Age at death<br>GLM quasipoisson because overdispersed with log link	Intercept	2.43	0.02	104.8	<0.001	Intercept	-1.08	0.53	-2.04	0.04
	relEV	0.001	0.0009	1.81	0.24	relEV	0.002	0.0009	2.30	0.02
						JL	0.01	0.002	6.68	<0.001

Supplementary Table S4. Summary pedigree statistics derived using the R package pedantics [33]. F refers to Wright's inbreeding coefficient.

Records	1715
Maternities	1241
Paternities	1088
Full sibs	60
Maternal sibs	1573
Maternal half sibs	1513
Paternal sibs	3632
Paternal half sibs	3572
Maternal grandmothers	736
Maternal grandfathers	667
Paternal grandmothers	380
Paternal grandfathers	390
Maximum pedigree depth (number of generations)	7
Founders	456
Mean maternal sibship size	2.44
Mean paternal sibship size	3.78
Non-zero F	55
F>0.125	1.00
Mean pairwise relatedness	0.005
Pairwise relatedness >=0.125	0.02
Pairwise relatedness >=0.25	0.007
Pairwise relatedness >=0.5	0.002

Supplementary Table S5. Full model outputs (R package: lmerTest, function: lmer): the significant variables were kept and formed the reduced models upon which the animal models (Table 1) were run.

Variable	Estimate	Standard Error	t	p
Model 1: all ages (n=488)				
Intercept (females, age 0, mother's location at parturition: Intermediate, mother's reproductive status: milk/winter yeld)	132.25	34.46	3.83	<0.001
Males	15.13	2.31	6.55	<0.001
Age 1	7.67	4.40	1.74	0.08
Age 2	17.27	7.18	2.40	0.02
Ages 3+	23.76	8.22	2.89	0.004
Jaw length	0.68	0.10	6.98	<0.001
Birth weight	2.83	0.96	2.94	0.003
Birth day in season	-0.11	0.08	-1.47	0.14
Mother's age at parturition	-0.20	0.38	-0.52	0.60
Mother's jaw length	-0.06	0.13	-0.51	0.61
Mother's location at parturition:				
Laundry greens	4.87	5.35	0.91	0.36
Mid glen	-4.52	3.93	-1.15	0.25
North glen	-7.50	3.52	-2.13	0.03
South glen	-6.16	5.28	-1.17	0.24
Shamnan Insir	-4.33	3.76	-1.15	0.25
Mother's reproductive status:				
Naïve	-0.14	3.34	-0.04	0.96
Summer/true yelds	2.72	2.43	1.12	0.26
Random effects:	*Variance*	*Std. Deviation*		
Birth year	9.54	3.09	NA	NA
Mother's ID	97.26	9.86	NA	NA
Residual	375.46	19.38	NA	NA
Model 2: adults only (n=249)				
Intercept (females, age 0, mother's location at parturition: Intermediate, mother's reproductive status: milk/winter yeld)	101.72	53.16	1.91	0.06
Males	19.49	4.14	4.71	<0.001
Age	0.29	0.43	0.67	0.50
Jaw length	0.69	0.15	4.57	<0.001
Birth weight	1.02	1.44	0.71	0.48
Birth day in season	0.02	0.12	0.20	0.84
Mother's age at parturition	-0.56	0.57	-0.98	0.33
Mother's jaw length	0.14	0.19	0.73	0.47
Mother's location at parturition:				
Laundry greens	10.05	8.74	1.15	0.25
Mid glen	-1.82	5.27	-0.35	0.73
North glen	-7.24	5.35	-1.35	0.18
South glen	-5.21	7.28	-0.72	0.48
Shamnan Insir	1.04	5.70	0.18	0.86
Mother's available investment:				
Naïve	5.67	4.95	1.15	0.25
Summer/true yelds	9.41	3.56	2.64	0.009
Random effects:	*Variance*	*Std. Deviation*		
Birth year	5.06	2.25	NA	NA
Mother's ID	121.94	11.04	NA	NA
Residual	384.32	19.60	NA	NA

Supplementary Table S6. Summary statistics of endocranial volumes in relation to sex and age. Juveniles: 0-2 years, adults: 3+ years; SD: Standard Deviation, CV: coefficient of variation.

Age	Sex	n	Mean (ml)	SD (ml)	CV
Juveniles	Females	213	261	30	11.58
	Males	303	276	33	12.12
Adults	Females	336	328	25	7.96
	Males	277	356	26	7.36

Supplementary Table S7. Offspring juvenile survival rate and endocranial volume. GLM: LRS as a proportion of LBS as the response variable and endocranial volume and jaw length as fixed effects with a binomial distribution and logit link (SE: Standard Error, z: z statistic, p: p-value).

Variable	Estimate	SE	z	p
Intercept	-4.44	1.34	-3.32	<0.001
Endocranial Volume	0.002	0.002	1.22	0.22
Jaw length	0.01	0.005	2.54	0.01

Dominance rank

We used dominance ranks from [32], which were calculated following [47] using data from 1974-1995. Dominance data only exist for females. Intra-cohort rank was controlled for age to distinguish among dominance and developmental effects and calculated as follows: the number of unrelated females of equal age or older that a focal individual threatened or displaced + 1 divided by the number of unrelated females of equal age or younger that a focal individual threatened or displaced + 1. Intra-cohort rank was then divided by the number of females in the cohort for a final score between 0 and 1 with higher numbers being more dominant.

There was very little dominance rank data, therefore a separate model on this small dataset (n=51 all ages, n=49 adults) was run to determine whether dominance rank or mother's dominance rank should be included in the reduced model. The full model was the same as above except sex was removed because no males had rank data, and dominance rank and mother's dominance rank were added as fixed effects. Dominance rank and mother's dominance rank were not significant variables in this version of the full model, therefore they were not included in the reduced models (Supplementary Table S8).

Table S8. Full model outputs showing that dominance rank and mother's dominance rank were not variables to include in the reduced models for the animal model.

Variable	Estimate	Standard Error	t	p
All ages model (n=51)				
Intercept (mother's location at parturition: Intermediate, mother's reproductive status: milk/winter yeld)	179.70	113.61	1.58	0.12
Ages 3+	-27.29	18.89	-1.45	0.15
Jaw length	1.11	0.21	5.22	<0.001
Birth weight	-3.67	2.89	-1.27	0.21
Birth day in season	0.05	0.37	0.14	0.89
Dominance rank	-3.39	11.09	-0.31	0.76
Mother's dominance rank	-6.54	3.42	-1.91	0.07
Mother's age at parturition	-1.26	1.57	-0.81	0.42
Mother's jaw length	-0.13	0.39	-0.33	0.74
Mother's location at parturition:				
Laundry greens	-26.67	24.23	-1.10	0.28
Mid glen	-17.49	10.03	-1.74	0.09
North glen	-2.18	9.93	-0.22	0.83
Shamnan Insir	-12.45	10.98	-1.13	0.26
Mother's reproductive status:				
Naïve	6.33	9.96	0.64	0.53
Summer/true yelds	11.13	6.88	1.62	0.11
Random effects:	*Variance*	*Std. Deviation*		
Birth year	116.00	10.77	NA	NA
Mother's ID	154.00	12.41	NA	NA
Residual	156.50	12.51	NA	NA
Adult only model (n=49)				
Intercept (mother's location at parturition: Intermediate, mother's reproductive status: milk/winter yeld)	147.92	124.66	1.19	0.25
Age	0.44	0.77	0.57	0.57
Jaw length	1.06	0.24	4.25	<0.001
Birth weight	-3.58	2.94	-1.22	0.23
Birth day in season	0.11	0.39	0.28	0.78
Dominance rank	-2.42	11.44	-0.21	0.83
Mother's dominance rank	-6.36	3.57	-1.79	0.09
Mother's age at parturition	-1.21	1.60	-0.75	0.45
Mother's jaw length	-0.11	0.40	-0.27	0.79
Mother's location at parturition:				
Laundry greens	-32.02	25.99	-1.23	0.23
Mid glen	-18.67	-18.67	-1.79	0.09
North glen	-3.19	10.31	-0.31	0.76
Shamnan Insir	-13.49	11.84	-1.14	0.26
Mother's reproductive status:				
Naïve	7.09	10.24	0.69	0.49
Summer/true yelds	12.07	7.06	1.71	0.09
Random effects:	*Variance*	*Std. Deviation*		
Birth year	119.30	10.92	NA	NA
Mother's ID	150.50	12.27	NA	NA
Residual	167.10	12.93	NA	NA

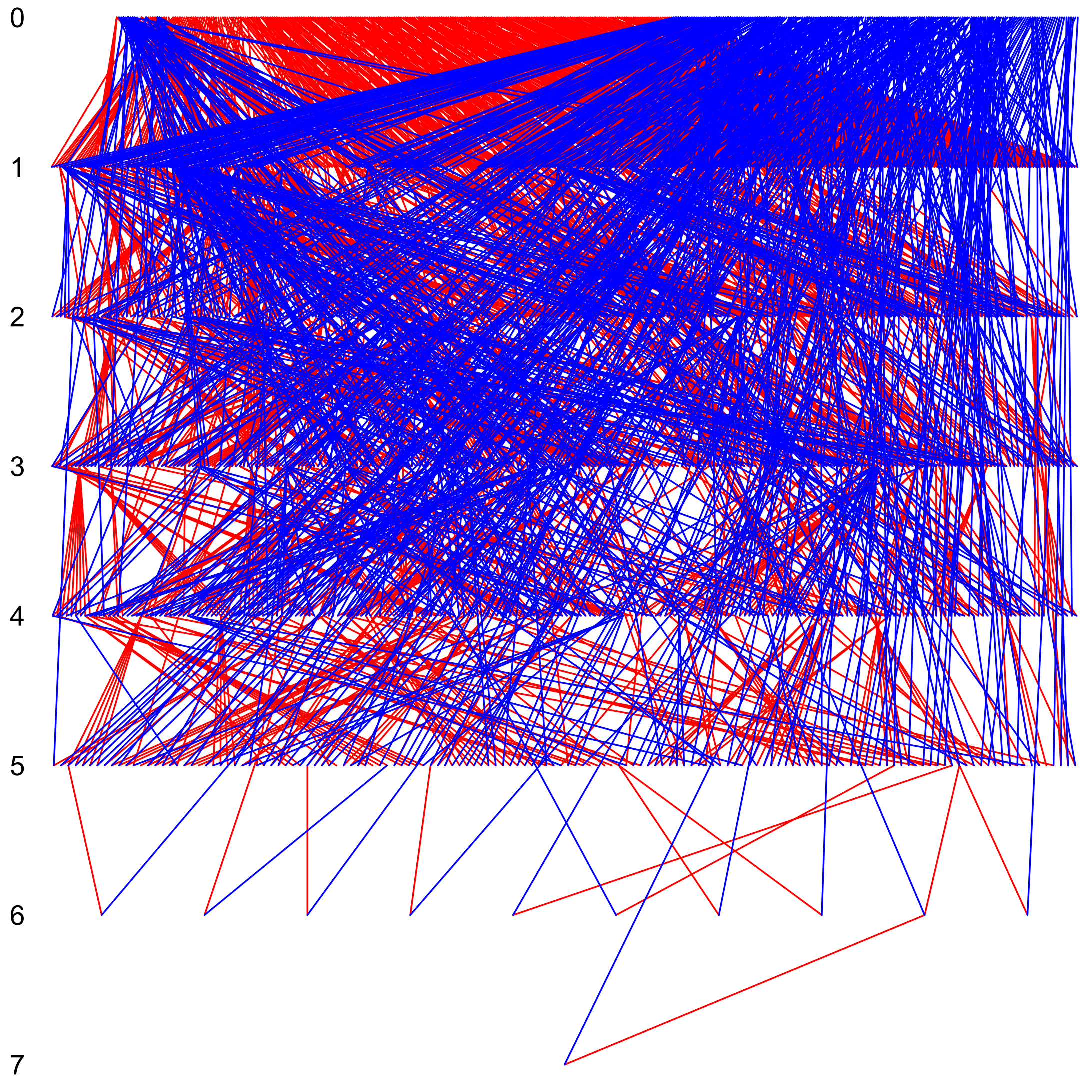

Supplementary Figure S1. The pedigree pruned to the deer whose endocranial volumes were measured (red lines = maternities, blue lines = paternities) and their relevant relatives. Each row represents one generation, from 0 through 7.

Further analyses of age-related variation in endocranial volume

We graphically compared endocranial volume vs age curves for individuals that died because they were shot outside the study area (n=129 females, n=178 males), which are a more random sample, with those that died of natural causes (n=493 females, n=489 males) in an initial analysis to ensure that the causes of natural deaths did not bias the results (R package: FlexParamCurve, function: pn.mod.compare [48]; R package: lattice, function: xyplot [49]). The curves for non-shot and shot deer were not different from each other (Supplementary Figure S2). Therefore, we can reliably determine adult status using all data (curves of best fit: female shot=richardsR31.lis, non-shot=richardsR12.lis; male shot=richardsR11.lis, non-shot=richardsR12.lis).

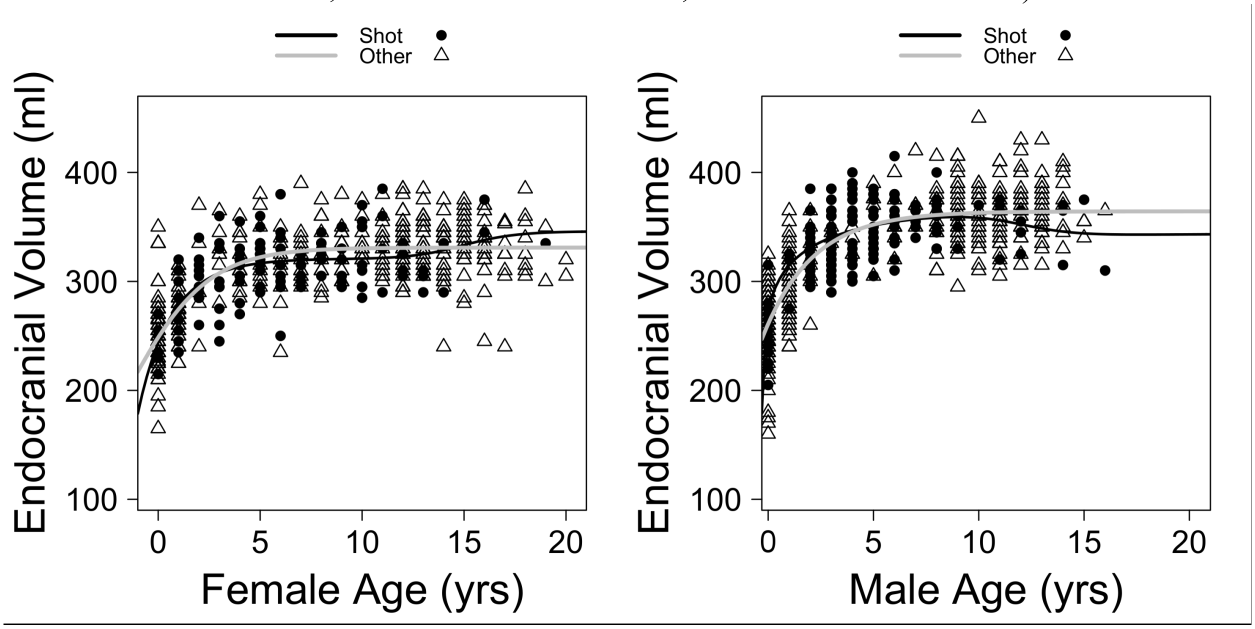

Supplementary Figure S2. Absolute endocranial volume by age in females (A) and males (B) that died of natural causes (filled circles, black line) or because they were shot (open triangles, light gray line).

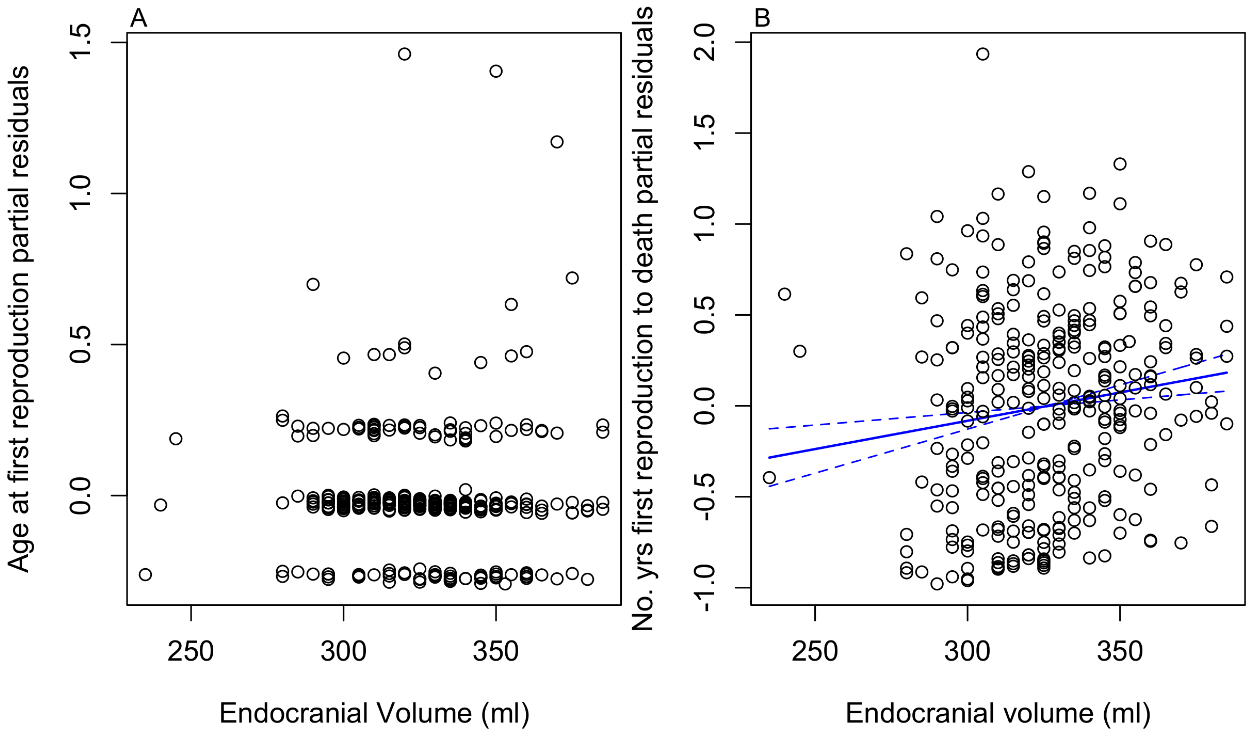

Supplementary Figure S3. Adult female (3+ years) age at first reproduction was not associated with relative endocranial volume (A), whereas those females with larger relative endocranial volumes lived longer from age at first reproduction to death (B). Relative endocranial volume = residuals of endocranial volume against jaw length, shaded region = 95% Bayesian credible intervals, see Table 3 for analyses).

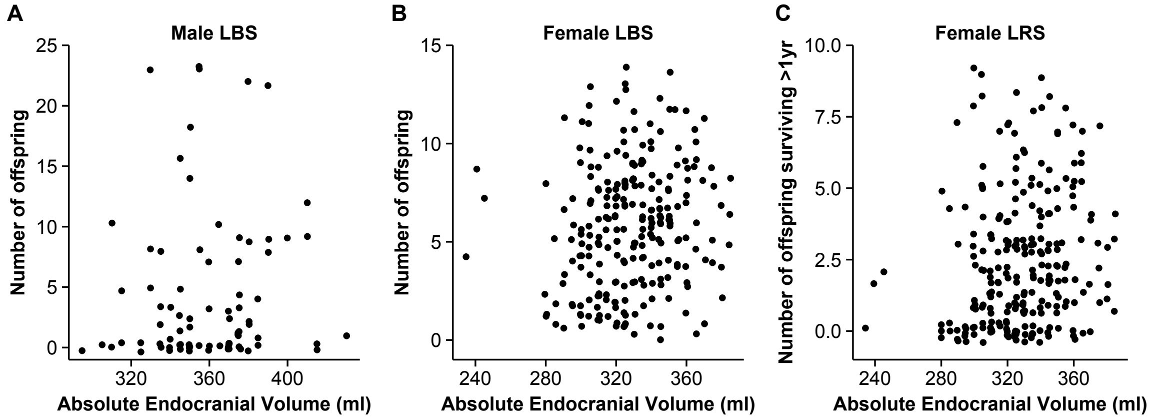

Supplementary Figure S4. Lifetime breeding (A, B) and reproductive success (C) for absolute endocranial volume in adults (3+ years) without accounting for jaw length.

References

47. Clutton-Brock TH, Albon SD, Guinness FE. 1986. Great expectations: dominance, breeding success and offspring sex ratios in red deer. *Anim Behav* **34**, 460-471. (doi:10.1016/S0003-3472(86)80115-4)
48. Oswald SA, Nisbet IC, Chiaradia A, Arnold JM. 2012. FlexParamCurve: R package for flexible fitting of nonlinear parametric curves. *Methods Ecol Evol* **3**:1073-1077. (doi:10.1111/j.2041-210X.2012.00231.x)
49. Sarkar D. 2008. Lattice: Multivariate Data Visualization with R, Springer. http://lmdvr.r-forge.r-project.org/. Accessed 3 January 2016.
